# Turning wood waste into high-value nanocellulose for sustainable agriculture environmental remediation and wound healing applications

**DOI:** 10.1038/s41598-026-69470-x

**Published:** 2026-09-15

**Authors:** Eman Tawfik, Nada Osama, Mohamed Tayel

**Affiliations:** 1 Botany and Microbiology Department, Faculty of Science, Capital University, Cairo, Egypt; 2 Chemistry Department (Biotechnology), Faculty of Science, Capital University, Cairo, Egypt

**Keywords:** Wood waste, Sustainable agriculture, Water retention, *Hordeum vulgare*, Metabolites, RAPD-PCR, SCoT marker, Bioremediation, Wound healing, Biological techniques, Biotechnology, Environmental sciences, Plant sciences

## Abstract

Wood processing industries generate staggering volumes of sawdust and residues. This research created a new way to turn wood waste into valuable resources, solving both environmental problems and agricultural challenges. Through step-by-step valorization pathways, sawdust was reused as a soil enhancer and processed into functional cellulose particles using chemical treatments—alkali digestion, peroxide bleaching, and acid hydrolysis. Characterization tests included: Zeta potential and size, Scanning electron microscope (SEM) and FT-IR. The zeta size showed nanocellulose populations at 95.82 nm and submicron cellulose (412.0 nm), with FT-IR spectroscopy proved they kept their natural structure by showing distinct hydroxyl (3339 cm⁻¹), hydrocarbon (2926 cm⁻¹), and glycosidic bond (1031 cm⁻¹) signatures. Scanning electron microscopy visualized the morphological transition from raw wood shavings to nano/micro-scale cellulose fibrils. This study used wood waste in four applications: water conservation, agriculture enhancement, bioremediation and medical wound healing. Sawdust reveals high water conservation within its particles due to high porosity on its surface. In agronomic trials with barley plant, the soil mixed with cellulose particles (S + C) significantly enhanced root development (11.5 cm) while keeping robust shoot elongation (23.5 cm), though sawdust-enriched soil (S + W) unexpectedly contained the highest leaf protein concentration (149.14 µg/mL). Genetic analysis confirmed all treatments induced low DNA variants, proving genomic safety. The polymorphism percentage from RAPD-PCR was 22.5%, while the polymorphism of SCoT-PCR was 13.7%. The extracted cellulose particles worked well in environmental remediation, reducing synthetic dye concentrations, and accelerated wound healing in cellular models. This work serves to achieve about seven goals of Sustainable Development Goals (SDGs) related to food, health, environment and climatic changes: SDG 2, 3, 6, 9, 12, 13 and 15.

## Introduction

 Wood-processing businesses around the world produce huge amounts of trash, like sawdust, shavings, and bark. It’s estimated that they produce around 1.2 billion tons of rubbish every year, and 20–66% of the wood that is processed ends up as waste during milling and harvesting. These wastes are typically burned, landfilled, or utilized for low-value products like mulch or biofuel, which puts a significant strain on the environment and increases CO_2_ emissions. Waste production rises in tandem with the world’s need for lumber and wood-derived bioproducts, creating sustainability issues and highlighting the pressing need to transition to high-value valorization techniques within the context of a circular economy^1,2^.

In response, Egyptian researchers are at the forefront of turning wood and agricultural waste into commodities with added value. For instance, particleboards with mechanical performance on par with traditional wood-based panels have been successfully made from sugarcane bagasse, offering a sustainable substitute for imported wood and lessening the strain on natural forests. Additionally, research shows that sawdust may be chemically processed and recycled into cellulose nanofibrils, which improve the microstructure and crack resistance of concrete and strengthens cement composites. Other initiatives include mixing plastics and wood residues to make wood-plastic composites, which repurpose polymer and agricultural waste and lessen the need for landfills while fostering the growth of regional industries. These developments have the potential to significantly reduce environmental burdens and support sustainable industrial growth, which is in line with Egypt’s circular economy objectives^3,4^.

The main component of biomass waste is cellulose, and its effective and highly valuable use could pave the way for a promising future in the use of biomass wastes. Cellulosic material with a single dimension in the nanometer range is called nanocellulose, and its primary sources include bacteria, plants, and mammals. The extraction of nanocellulose from various biomass sources for use in a range of fields has drawn more interest recently^5^. Cellulose nanocrystals (CNCs), also known as nanocrystalline celluloses (NCCs), nanocrystalline cellulose, nanocellulose whiskers (CNWs), and rodlike cellulose microcrystals, are the three main subcategories of nanocellulose. The former is primarily made by acid hydrolyzing lignocellulosic materials to remove amorphous cellulose and leave behind rod-like cellulose nanocrystals. (2) Cellulose nanofibers (CNFs/NFCs), also known as micro-fibrillated cellulose (MFC), nanofibrils, or microfibrils, create cellulosic fibers by mechanical processing (or in conjunction with chemical or enzymatic pretreatments). (3) Several species of Acetobacteraceae are the primary producers of bacterial cellulose (BC), also known as bacterial nanocellulose (BNC), microbial cellulose, and bio-cellulose^2,6^.

When added to soil, nanocellulose fibers made from wood waste effectively retain water due to their high porosity and hydrophilicity. Their structure at the micro- to nanoscale boosts the soil’s ability to hold water, which lowers the frequency of irrigation and promotes sustainable agriculture. Nanocellulose-amended soils have demonstrated enhanced root penetration and shoot growth in agronomic research. For example, TEMPO-oxidized wood pulp’s cellulose nanofibrils created hydrogels with a high swelling capacity that helped barley and other crops absorb nutrients and retain moisture^7^.

Nanocellulose’s large surface area and customizable surface chemistry make it an effective adsorbent for dye and pigment removal in wastewater treatment. Functionalized forms, such as carboxylated or amine-modified nanocellulose, bind positively charged dyes or heavy metals through electrostatic attraction or chelation. For example, TEMPO-oxidized nanocellulose containing amino or thiourea groups effectively adsorbed reactive colors and heavy metal ions from model effluents. Hybrid nanocellulose-metal oxide photocatalytic composites (e.g., TiO₂ or ZnO) can destroy organic pigments under UV/visible light, providing a two-pronged approach—adsorption and photodegradation to filter water more effectively^8^.

Applications in tissue engineering, tissue repair, and wound healing are further significant uses of nanocellulose that are covered in greater detail in the section that follows. Blood vessel, neural, bone, cartilage, liver, and adipose tissue engineering, urethral and dura mater reconstruction, connective tissue repair, congenital heart defect repair, protective barrier construction, and ophthalmologic applications—primarily contact lens construction—are the primary examples of these applications^7,9^. Nanocellulose from wood pulp has great biocompatibility, non-toxicity, high porosity, and moisture retention, making it perfect for wound dressings. Recent research has created multifunctional nanocellulose-based hydrogels containing antimicrobial agents such as silver nanoparticles, curcumin-stabilized AgNPs, or antibiotics, which have strong antibacterial action against pathogens (e.g., *S. aureus*, *E. coli*) and promote haemostasis. In vivo animal studies (e.g., rat/mouse) have demonstrated rapid wound closure, angiogenesis, and tissue regeneration, highlighting wood-derived nanocellulose as a viable platform for enhanced wound dressing and tissue engineering applications^10,11^.

This project aims to create a comprehensive, sustainable method for valorizing wood waste, particularly sawdust, by transforming it into functional nanocellulose and assessing its diverse applications in agriculture, environmental remediation, and medicinal treatment. The study aims to (1) enhance the extraction and characterization of nanocellulose from wood byproducts, (2) evaluate its effects on soil characteristics and plant growth (utilizing barley plant as a model), (3) explore its capacity for dye bioremediation, and (4) assess its efficacy in facilitating wound healing, all in alignment with significant sustainable development goals (SDGs).

## Materials and methods

### Wood waste materials

The term “wood waste” in this study refers to untreated lignocellulosic leftovers from woodworking operations, mainly sawdust and fine wood shavings. This substance was utilized directly in the experimental applications as indicated and as the raw feedstock for the extraction of nanocellulose. Sawdust and fine wood shavings made up the majority of the wood waste that was gathered from a nearby woodworking workshop in Cairo, Egypt. Paints, adhesives, and chemical preservatives were not present in the debris, which was made from mixed untreated wood scraps produced during cutting and carpentry operations. Before being used in the nanocellulose extraction procedure, the material was air-dried, ground into a uniform fine powder, and sieved to get rid of big particles.

### Preparation of nanocellulose

To achieve a uniform particle size, the gathered wood waste was air-dried, mechanically ground into a fine powder, and then run through an appropriate screen. Following dry storage, the resulting wood powder was utilized to extract nanocellulose. The nanocellulose was prepared according to Kargarzadeh et al.^12^, as follow: The dried fine powder of wood waste was combined with 17.5% NaOH and heated at 80–90 °C for 2–3 h while stirring. Filter and rinse with distilled water until achieving a neutral pH. Combine the residue with a 1:1 mixture of H₂O₂ and NaOH, heat to 60–70 °C for 1–2 h, and subsequently wash until neutral. Subsequently, acid hydrolysis was conducted by incorporating 64% H₂SO₄ into the cellulose pulp at a solid-to-liquid ratio of 1:10. Stir at 45 °C for 30 to 60 min in an ice bath to regulate temperature and avert deterioration. Centrifuge at 10,000 revolutions per minute for 10 min, then discard the supernatant. Continue washing and centrifugation until the pH reaches around 5–6. Conduct dialysis with distilled water until achieving a neutral pH or perform complete centrifugation again. Employ an ultrasonicator for 30 to 60 min or a high-shear mixer to disperse and diminish the size of nanocellulose.

### Characterization of wood and nanocellulose

#### Scanning electron microscope (SEM)

Scanning Electron Microscopy (SEM) is a powerful technique for characterizing particles, providing detailed information about their morphology, size, and elemental composition. SEM uses a focused electron beam to scan the sample’s surface, generating signals that reveal information about the particle’s topography and composition. By analyzing these signals, SEM can produce high-resolution images and perform microanalysis, making it a versatile tool for particle analysis^13^. The morphology of the extracted nanocellulose was examined using a JEM-1010 transmission electron microscope (JEOL Ltd., Tokyo, Japan). The obtained micrographs were used to evaluate the size and morphological characteristics of the nanocellulose particles.

#### FT-IR of wood and nanocellulose

FT-IR (Fourier Transform Infrared) spectroscopy is a powerful technique used to characterize the chemical composition of particles. The intensity of the absorbed light at different wavelengths is measured and converted into a spectrum, which is a unique fingerprint of the material’s chemical composition^14^. (FTIR) spectra of the wood waste and extracted nanocellulose were recorded using a Bruker Tensor 27 FTIR spectrometer (Bruker Optics GmbH, Ettlingen, Germany).

### Zeta sizer for nanocellulose

The zeta potential of nanocellulose was assessed using a Malvern Zetasizer Nano ZS by laser Doppler micro-electrophoresis. Aqueous suspensions of nanocellulose (~ 0.1–0.5 mg/mL) were formulated in deionised water and subjected to sonication for 1–2 min to achieve uniform dispersion. The suspensions were placed onto a clean folded capillary cell (DTS1060C), and measurements were performed at 25 °C. The instrument settings comprised an applied voltage of approximately 40 V, with 50 to 100 sub-runs per sample, utilizing the Smoluchowski model to translate electrophoretic mobility into zeta potential. A minimum of three replicate measurements were conducted for each sample to guarantee repeatability. Data quality was assessed using a mean count rate (≥ 10 kcps) and a Quality Factor (> 1), with results shown as mean ± standard deviation (e.g., − 34.3 ± 2.5 mV). This approach adheres to standardized standards for assessing the colloidal stability and surface charge of cellulose nanoparticles^15^. The zeta potential and particle size distribution were measured using a Malvern Zetasizer Nano ZS (Malvern Panalytical, Malvern, UK).

### X-ray crystallography of nanocellulose (XRD)

XRD patterns of the nanocellulose samples were recorded using a Cu Kα radiation source (λ ≈ 1.54 Å), typically generated at 40 kV and 40 mA, over a 2θ range of about 5–40° (commonly extended to 60–70° in some studies) at a scan rate around 1°/min. Diffractograms were used to identify the characteristic cellulose I reflections, with the (200) plane appearing near 22.6° and the overlapping (1–10)/(110) planes near 14.8–16.5°. Sample crystallinity was quantified using the empirical Segal method, CrI (%) = [(I002 – IAm)/I002] × 100, where I002 is the maximum intensity of the (200) peak near 22.6° and IAm is the minimum intensity of the amorphous halo near 18°^16^.

### Assessment of water retention utilizing wood, nanocellulose, and composite amendments

Soil samples (clay soil) are initially air-dried, sieved, and categorized into five treatments: (1) soil only (5 g), (2) wood sawdust (5 g), (3) soil + wood sawdust (2.5 g + 2.5 g), (4) soil + nanocellulose fibers (2.5 g + 2.5 g), (5) nanocellulose (5 g). Each mixture is homogenized and transferred into filter paper on funnel for testing. A 50 ml of dH_2_O was poured on each funnel and left for about 1 h till complete drainage of water. Gravimetric water content is determined by weighing the drainage water, also the water retained within particle of each funnel was recorded beside time taken for drainage^17^. All experiments were performed using the same batch of air-dried and sieved clay soil under controlled laboratory conditions (25 ± 2 °C), and each treatment was conducted using three independent biological replicates.

### Application of wood and nanocellulose in soil

A100 gm of soil was weighted and mixed with sawdust, nanocellulose and mix of them, comparing to control clay soil only. Then, 20 grains of barley and cultivated in each pot for 28 days with regular irrigation of water. The barley was for Giza-132 cultivars brought from Agriculture Research Center, Giza, Egypt. Then record morphological and physiological parameters, then estimate genetic variation.

### Morphological parameters

The morphological description for the growth parameters reflects the response of barley species to grow in soils supported by different parameters like wood waste, nanocellulose and their mixture in clay soil. Five parameters were estimated: fresh weight, shoot length, leaf length, number of roots, root length.

### Physiological parameters

The physiological parameters reflect how the internal metabolic reactions acclimatize to the change in soil composition and addition of wood waste or cellulose to soil. The measured physiological parameters were: chlorophyll index, total proteins, total phenolics and total flavonoids. All these protocols were performed according to protocols mentioned in Bahnasy et al.^18^.

### Genetic variation

#### DNA extraction from barley leaves

Young barley leaves were harvested and promptly frozen in liquid nitrogen, thereafter, pounded into a fine powder using a mortar and pestle or bead mill. Approximately 50–60 mg of frozen tissue was utilised per sample, and complete genomic DNA was extracted with the CTAB process as described by Doyle and Doyle^19^, incorporating CTAB buffer, chloroform–isoamyl alcohol purification, and ethanol precipitation. The purity and content of DNA were evaluated using spectrophotometry and agarose gel electrophoresis. Purified DNA was preserved at − 20 °C before PCR analysis.

#### Genotyping by RAPD‑PCR and SCoT‑PCR

Decamer primers (10 nucleotides) with roughly 50–60% GC content was utilized for RAPD analysis. PCR procedures (20–25 µL) comprised template DNA (~ 25–100 ng), 12.5 µL PCR master mix, and 2 µL of primer, resulting in a total volume of 25 µL. A standard thermal cycler protocol consisted of: initial denaturation at 95 °C for 5 min, followed by 35 cycles of denaturation at 95 °C for 30 s, low-temperature annealing at 35–37 °C for 30 s, extension at 72 °C for 40 s, and a final extension at 72 °C for 10 min. PCR results were visualized on 1.5% agarose gels, stained with ethidium bromide, and photographed.

Start Codon Targeted (SCoT) analysis employed gene-specific primers surrounding the ATG start codon to identify functional polymorphisms. A standard SCoT-PCR mixture comprised approximately 25 ng of DNA, 12.5 µl of PCR master mix, and 2 µl of primer, resulting in a total volume of 25 µl. The cycling conditions often included an initial denaturation at 95 °C for 5 min, succeeded by 35 cycles comprising denaturation at 94 °C for 30 s, annealing at about 58 °C for 30 s, extension at 72 °C for 1 min, and a concluding extension at 72 °C for 10 min^20,21^. The gels were analyzed in a manner analogous to RAPD products.

Presence/absence matrices of bands were produced from distinct and consistent banding patterns. The data were utilized to calculate genetic similarity indices and to visualize diversity through clustering methods, such as UPGMA dendrograms, facilitating the assessment of genetic variance among barley treatments using both RAPD and SCoT markers.

### Bioremediation of dyes

The third application of wood waste is the bioremediation of dyes, which is a method of cleaning up dye-contaminated wastewater and soil using wood waste and nanocellulose. Salazar-Rabago et al.^22^. developed a method involving sawdust for dye remediation as follow: weight 0.01 gm of different dyes in 100 ml of dH_2_O (malachite green, neutral red and bromophenol blue). Incubate 5 g of wood waste and nanocellulose in each dye solution for 3 h then measure absorbance to record the leaching of dyes.

### Wound healing bioassay

A wound healing assay is a medical and the fourth application for nanocellulose, also known as a scratch assay. It is a simple in vitro technique used to study wound closure due to treatment with nanocellulose compared to control wound without any additives^23^. A cell-free area is generated in a confluent cell layer of small mice, typically by scratching the monolayer with a pipette tip or using a specialized insert or stencil. Images are captured at intervals, and data on wound closure with dimensions were analyzed. The mice separated into two groups: treated with bandage with nanocellulose and control (bandage only) and the groups were repeated in triplicates. The average wight of each mouse is about 27 g. The authors have followed the rules of the ethical committee of Institutional Animal Care and Use Committee (HU-IACUC) Faculty of Science, Capital University, Approval Number: HU-IACUC/B/ET0709-02.

All animal experimental procedures were performed in accordance with the relevant institutional guidelines and regulations and complied with the ARRIVE guidelines for the reporting of animal experiments. All protocols were approved by the Institutional Animal Care and Use Committee (HU-IACUC), Faculty of Science, Capital University (Approval No. HU-IACUC/B/ET0709-02).

### Statistical analysis

On the gathered data, descriptive analysis of the variance test and one-way ANOVA were run in Minitab 19. Along with correlations and standard deviations, the mean average was estimated.

## Results

### Preparation and characterization of nanocellulose from sawdust

**Fig. 1:**
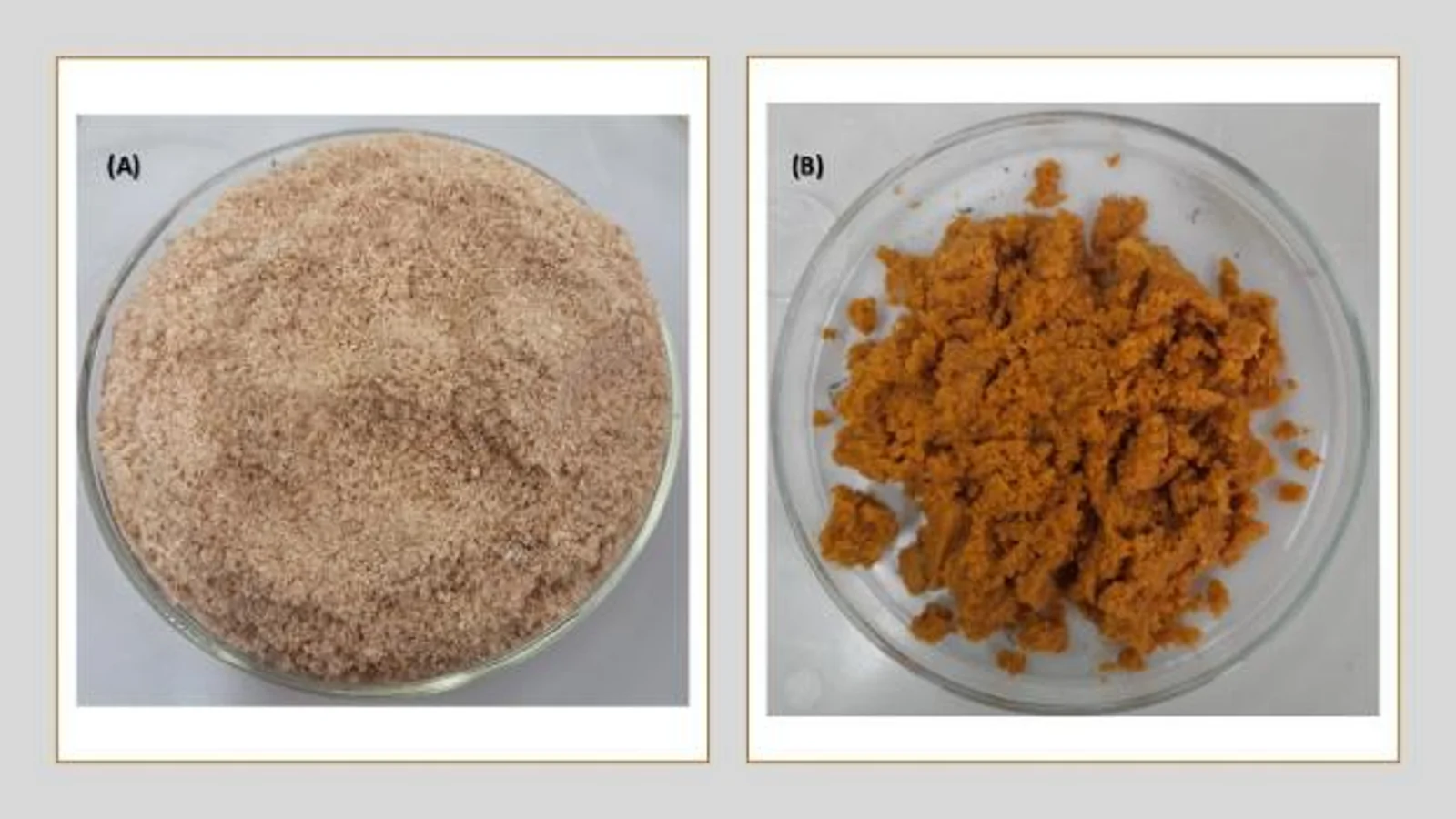
The different morphotypes of (**A**) Sawdust, (**B**) Nanocellulose.

The different morphotypes of (A) sawdust and (B) nanocellulose made from wood waste are depicted in Fig. 1. The dry, fine ground, light-beige sawdust particles in Fig. 1A are suggestive of untreated lignocellulosic material. This form has a small surface area and little reactivity, while maintaining the bulk structure of the original wood fibers. Figure 1B, on the other hand, displays nanocellulose following chemical treatment as an orange mass that is wet, dense, and fibrous. The substantial structural change brought about by procedures like alkali treatment, bleaching, and acid hydrolysis, which break down the lignin and hemicellulose matrix and reveal cellulose micro- and nanofibrils, is reflected in this change in morphology.

**Fig. 2:**
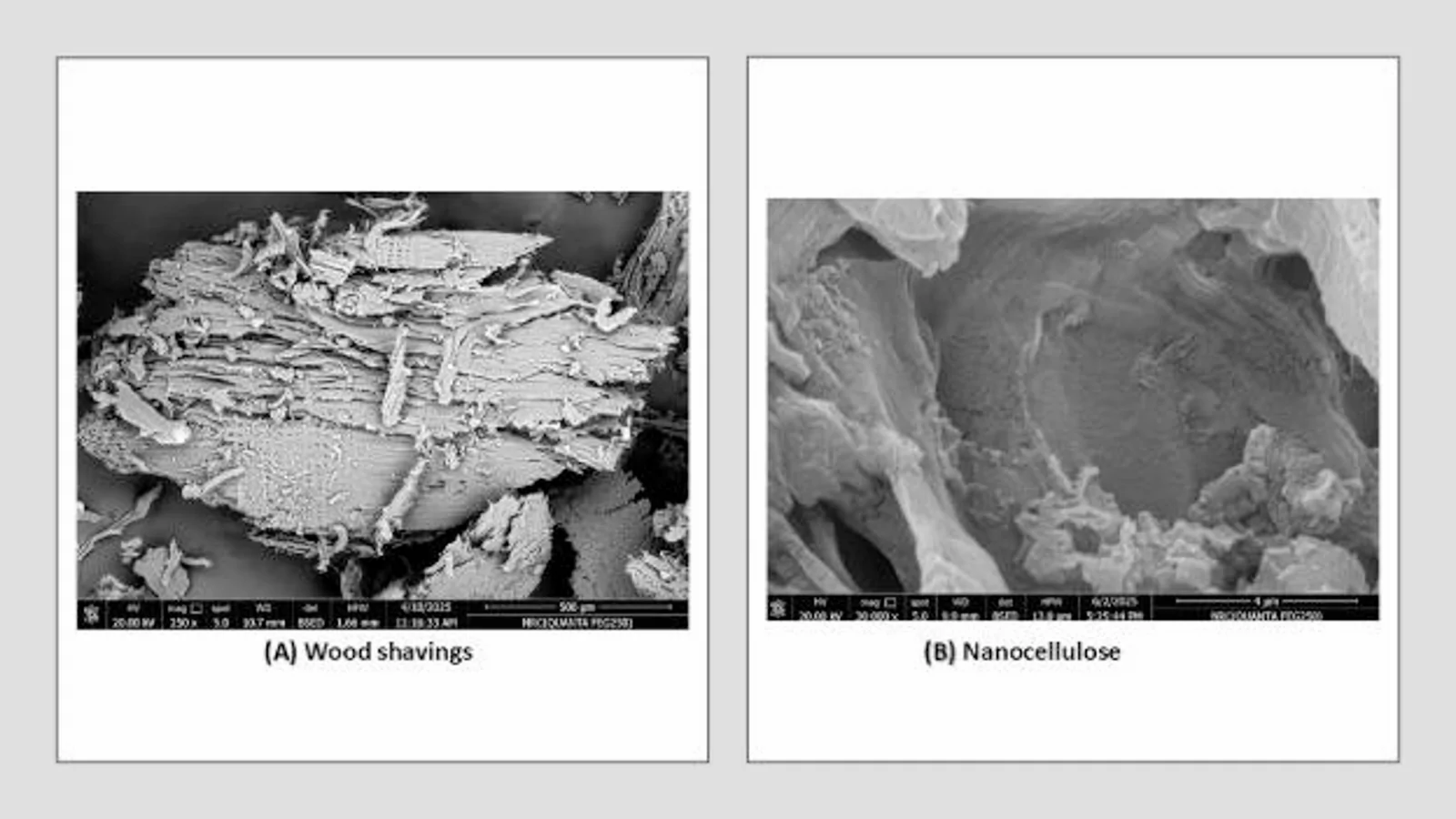
Scanning electron micrographs showing the morphological transition from raw wood to nanocellulose. (**A**) Raw wood shavings display large, rigid lamellar structures typical of untreated lignocellulosic biomass (magnification: 250×; scale bar: 500 μm). (**B**) Nanocellulose shows a fibrillated, porous structure with nanoscale features resulting from chemical processing (magnification: 30,000×; scale bar: 4 μm).

The scanning electron microscopy (SEM) images illustrate the morphological alteration of wood material during nanocellulose synthesis. Fig. 2A, unrefined wood shavings display a rough, flaky composition characterized by substantial, compressed cellulose layers and discernible fiber lamellae. The coarse texture and stratified structure are indicative of untreated lignocellulosic biomass, which has both cellulose and residual lignin. Conversely, Fig. 2B displays nanocellulose at a far higher magnification, elucidating a more refined, porous, and fibrillated shape. The disintegration into micro- and nanoscale fibrils is apparent, characterized by smoother surfaces and enhanced surface area, signifying effective chemical processing (e.g., alkali treatment and acid hydrolysis). This change improves the material’s functional attributes, including water retention, surface reactivity, and compatibility for bio-composites and biomedical applications. The disparity in scale bars (500 μm versus 4 μm) underscores the substantial size reduction and fibrillar refinement attained in nanocellulose manufacturing.

**Fig. 3:**
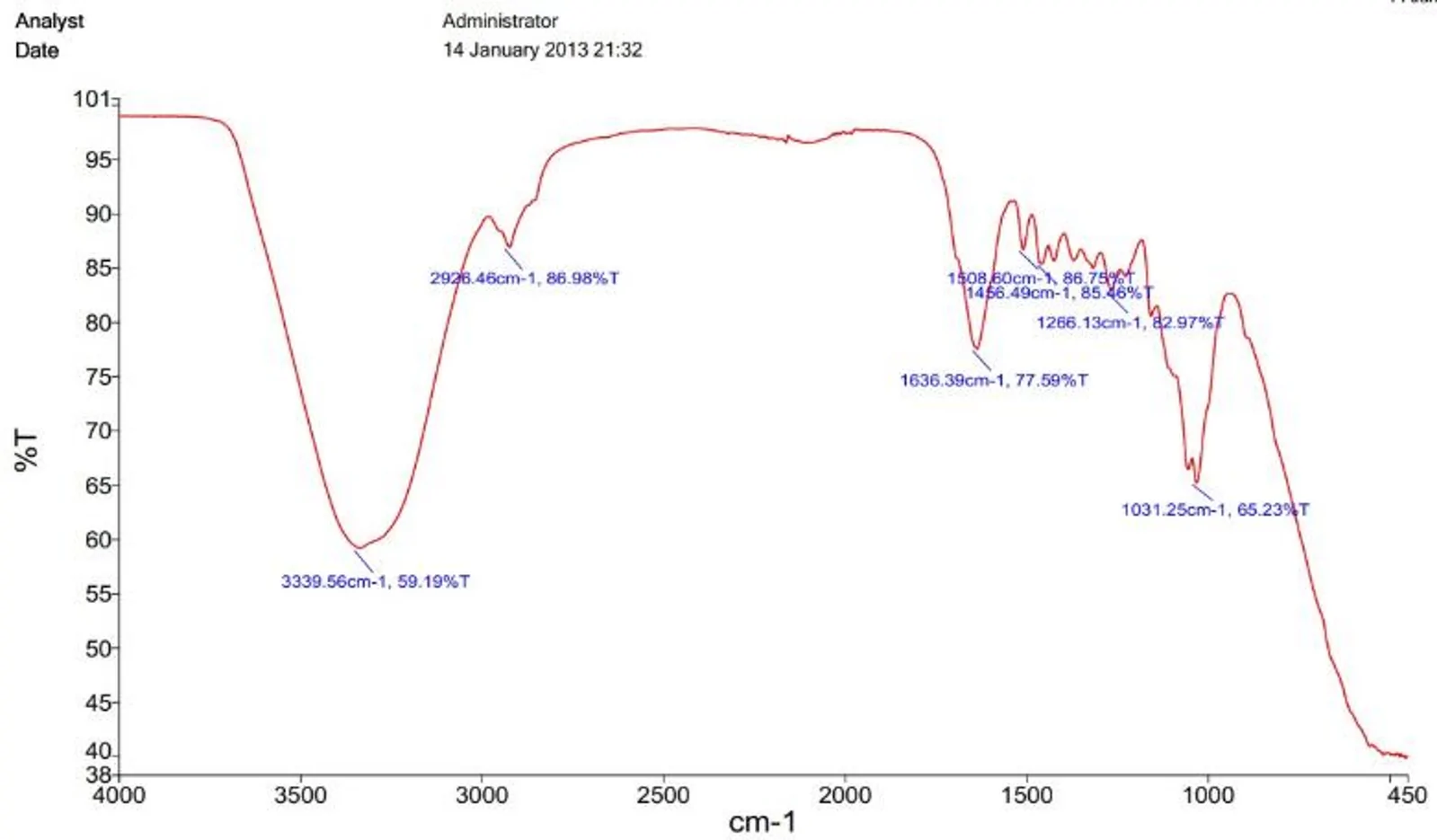
FT-IR analysis of nanocellulose particles extracted from sawdust.

The findings of the analysis of the nanocellulose’s FT-IR spectra are displayed in Fig. 3. According to these peaks exhibit different transmittance percentages (%T) at wavenumbers (cm-1). For example, there is a prominent peak at 1031.25 cm-1 with 65.23%T and another strong absorption at 3339.56 cm-1 with 59.19%T. These peaks’ existence and strength can be utilized to pinpoint functional groups in the CNS sample, revealing details on its molecular makeup.

**Fig. 4:**
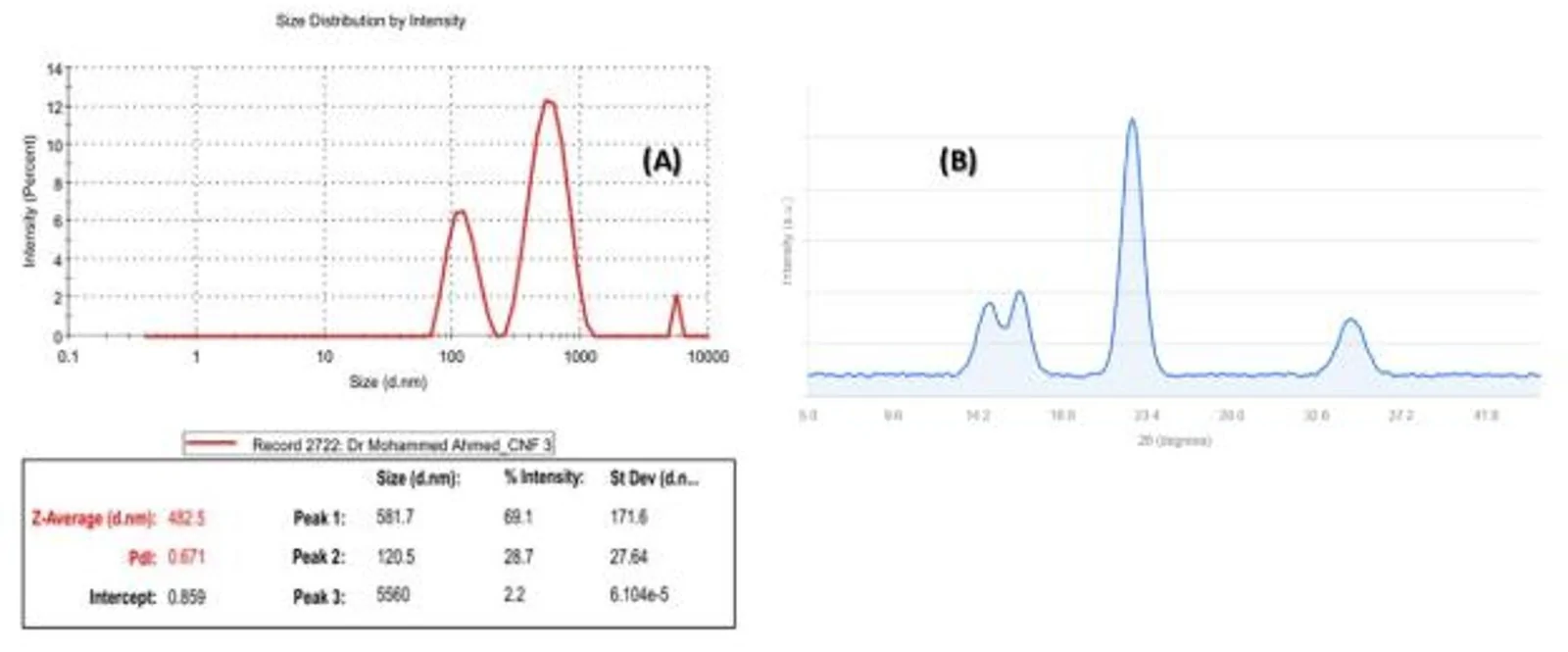
(**A**) Dynamic light scattering (DLS) size distribution by intensity of nanocellulose fibers. (suggesting the presence of nanofibrils, smaller nanocrystalline fragments, and a minor fraction of larger aggregates). (**B**) XRD of nanocellulose fibers.

Figure 4A, the size distribution of nanocellulose fibers by intensity, as determined using dynamic light scattering (DLS). The Z-average diameter is 482.5 nm, signifying the intensity-weighted mean hydrodynamic diameter of the suspended particles. Three separate summits are noted: Peak 1 at 581.7 nm (69.1% intensity) signifies the predominant nanocellulose component, whereas Peak 2 at 120.5 nm (28.7%) presumably pertains to smaller cellulose nanocrystals or fragmented fibrils. Peak 3 at 5560 nm (2.2%) signifies the existence of bigger aggregates or remnant microfibrils. The observations indicate that the nanocellulose preparation comprises both nano- and submicron-sized fractions, potentially affecting its utility in water retention, composite reinforcement, or biological applications. Figure 4B illustrated the native crystalline form of cellulose, which is usually retained in nanocellulose following the chemical isolation, has this XRD pattern. While the minor peaks at 14.8° and 16.5° originate from the overlapping (1–10) and (110) planes, the major peak at 22.6° belongs to the (200) crystallographic plane and serves as the main reference for calculating the crystallinity index. Diffraction along the cellulose chain axis is reflected by the much weaker peak at 34.5°, which is attributed to the (004) plane. The predicted crystallinity index using the Segal method on this pattern is roughly 91.7%.

### Water retention and gravitational waster estimation

**Fig. 5:**
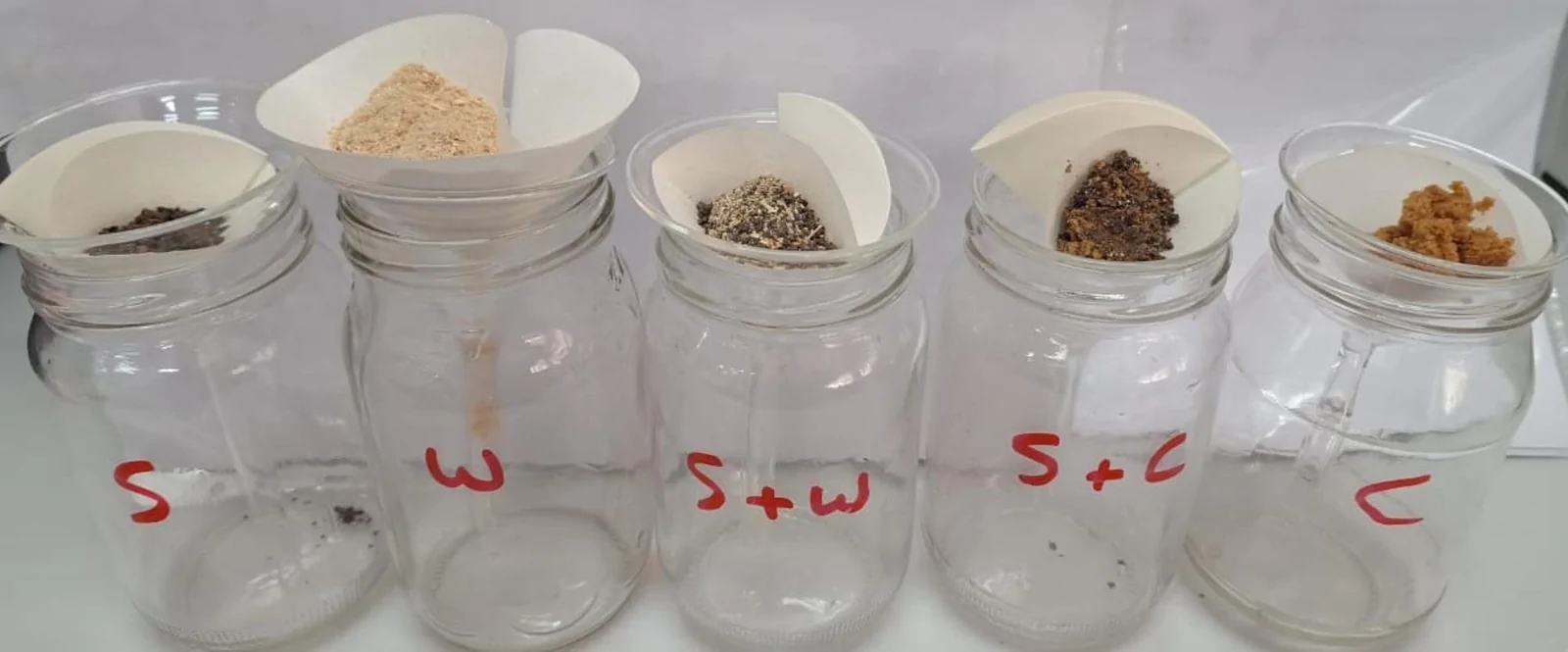
Water retention and gravimetric water estimation in different types of soil. (S: clay soil, W: sawdust, S + W: clay with sawdust, S + C: clay with nanocellulose, C: nanocellulose fibers).

It can be observed (in Fig. 5) that the structure of each sample seems to be quite uniform, devoid of filler aggregates or lumps of biopolymer. The good dispersion of the polymer and the fillers in the water solution further suggests that a good mixing method was applied.

Table 1 demonstrated the effects of several soil amendments: sawdust, nanocellulose, and their mixtures with clay; on water drainage duration and water retention capacity. Sawdust demonstrated the greatest water retention (26.21 mL) and the shortest drainage time (1.07 min), signifying quick water absorption and robust moisture retention owing to its elevated porosity and fibrous composition. Clay soil, albeit exhibiting a modest gravitational water capacity of 43 mL, held significantly less water at 5.81 mL and demonstrated an extended drainage time of 12.06 min, indicative of its hard texture and restricted internal porosity. The combination of clay and sawdust enhanced retention (10.23 mL) and decreased drainage time (1.52 min) relative to clay alone, hence validating the synergistic impact of integrating organic fiber with mineral soil. Nanocellulose exhibited intermediate drainage (6 min) but low retention (3.46 mL), likely attributable to its gel-like properties and smaller particle size, which restrict water entrapment during gravitational flow. Notably, the combination of clay and nanocellulose produced the longest drainage duration (17.53 min) yet exhibited unexpectedly low water retention (3.87 mL), maybe indicating fast leaching through the matrix or limited pore expansion. The results indicate that sawdust is more effective for water-holding applications, whereas nanocellulose largely influences flow dynamics rather than improving retention.

**Table 1:** Water drainage time, gravitational water, and water retained across different soil treatments. Each treatment was evaluated for time to gravitational drainage (in minutes), the volume of drained water (mL), and the amount of water retained within the matrix (mL).

Treatment	Time estimated (min)	Gravitational Water (ml)	Water retained (ml)
Caly soil only	12.06 ± 0.55^b^	43 ± 1.22^a^	5.81 ± 0.098^c^
Sawdust only	1.07 ± 0.022^d^	24 ± 0.074^c^	26.21 ± 1.25^a^
Clay + sawdust	1.52 ± 0.033^d^	39 ± 0.098^b^	10.23 ± 0.67^b^
Clay + nanocellulose	17.53 ± 0.67^a^	45 ± 1.33^a^	3.87 ± 0.033^c^
Nanocellulose only	6 ± 0.098^c^	42 ± 1.067^a^	3.46 ± 0.033^c^
P-value	0.001**	0.001**	0.001**
F-value	986	243	669

** highly significant.

### Application of wood and nanocellulose in soil

**Fig. 6:**
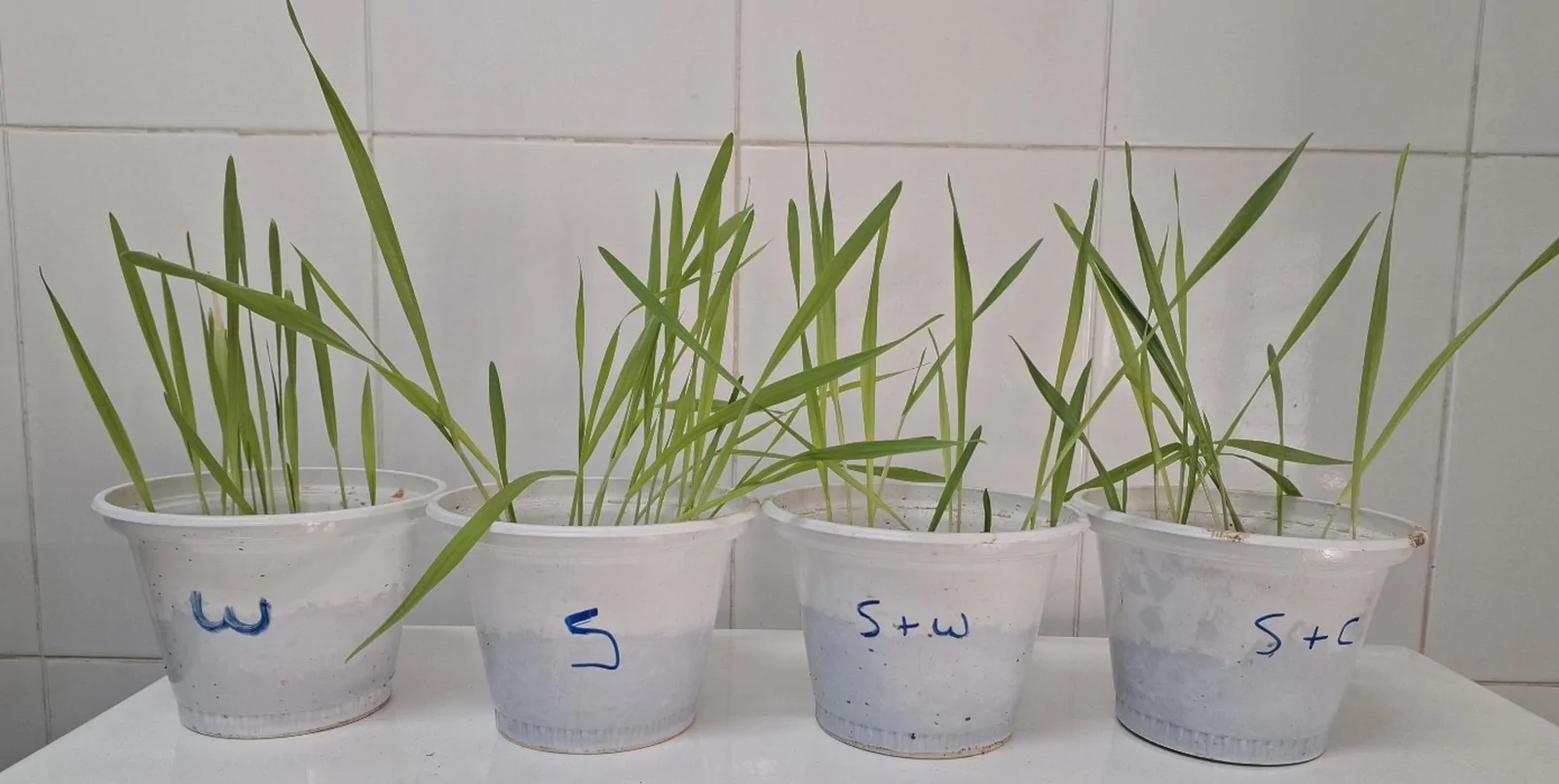
The germination of *Hordeum vulgare* in soil supplemented with wood and nanocellulose. W: sawdust only, S: clay soil only, S + W: clay soil with sawdust, and S + C: clay soil with nanocellulose.

**Fig. 7:**
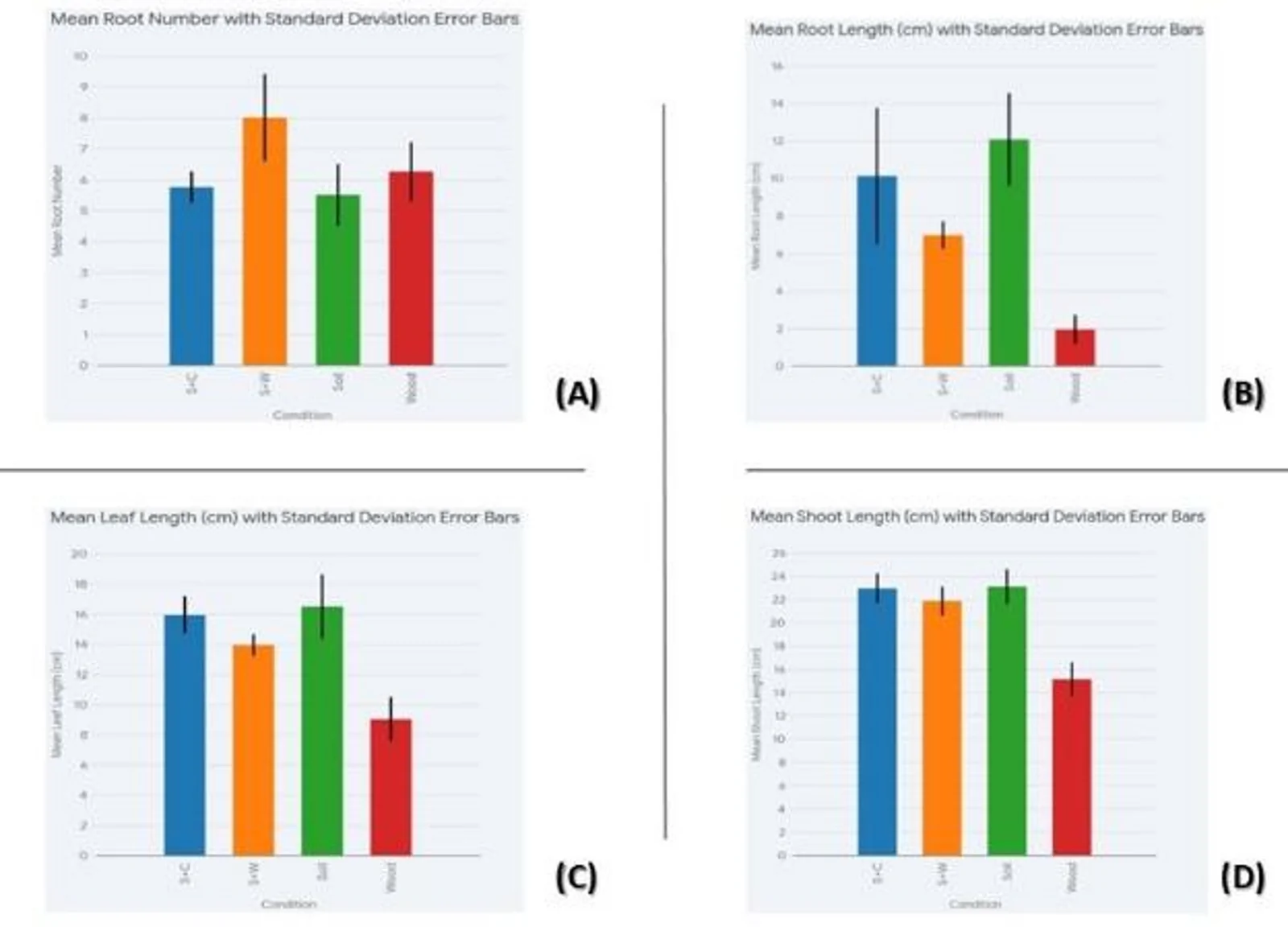
The morphological parameters of barley species cultivated in different soil natures. (green bar for clay soil only, red bar for sawdust wood only, orange bar for clay with sawdust, and blue bar for clay with nanocellulose).

The cultivated barley species in different soils were illustrated in Fig. 6. While Fig. 7 shows how the morphological traits of barley changed in response to four different types of soil: clay only, sawdust only, clay + sawdust, and clay + nanocellulose. The clay plus sawdust (S + W) condition had the most roots, followed by the wood only condition. This suggests that adding organic matter helps roots grow. When there was only clay soil, there were the fewest roots. Barley plants growing in clay + nanocellulose (S + C) and clay only had roots that were much longer than those grown in sawdust and wood. The roots were quite stunted in the wood-only condition, which suggests that pure sawdust may have caused compaction or inadequate aeration. The clay-only and clay + nanocellulose treatments made the leaves the longest, measuring about 16–18 cm. The wood-only treatments, on the other hand, made the leaves shorter, which again suggests that the conditions were not good for growth above ground. In S + C, S + W, and clay soil, the tallest shoots were always seen. On the other hand, wood-only treatment significantly slowed down shoot growth. This shows that combining cellulose or sawdust with clay helps shoots grow the best, but using sawdust alone may limit the growth of both roots and shoots.

### Physiological parameters

The physiological data show that clay + sawdust treatment greatly improved most of the biochemical features in barley (Table 2). This suggests that mixing organic matter with mineral-rich soil has a synergistic effect. This group had the most total protein (149.14 µg/g), phenolics (1.936 µg/g), and flavonoids (2.919 µg/g), as well as the highest chlorophyll index (3.8). This shows that nitrogen is more available and that secondary metabolites are made more easily. On the other hand, clay soil alone had the lowest total biochemical reactions (save for phenolics), which shows that unamended mineral soil can’t support barley’s best physiological growth. This shows how important it is to add biodegradable ingredients like sawdust and nanocellulose to soil to make it better at photosynthesis and more resistant to stress.

**Table 2:** Physiological parameters in barley plants grown in soils with sawdust, nanocellulose, and their composites.

Clay soil	Chlorophyll index	Total proteins (µg/g)	Total phenolics (µg/g)	Total flavonoids (µg/g)
	2.9 ± 0.22^b^	100.57 ± 3.55^c^	1.338 ± 0.05^b^	1.904 ± 0.05^c^
Sawdust soil	1.5 ± 0.13^c^	134.85 ± 4.67^b^	1.211 ± 0.04^c^	2.827 ± 0.06^ab^
Clay + Sawdust	3.8 ± 0.33^a^	149.14 ± 3.15^a^	1.936 ± 0.05^a^	2.919 ± 0.06^a^
Clay + Nanocellulose	2.7 ± 0.22^b^	106.71 ± 2.98^c^	1.027 ± 0.03^d^	2.698 ± 0.05^b^
P-value	0.001	0.001	0.001	0.001
F-value	48.3	119.7	247.4	212

### Molecular variation

**Fig. 8:**
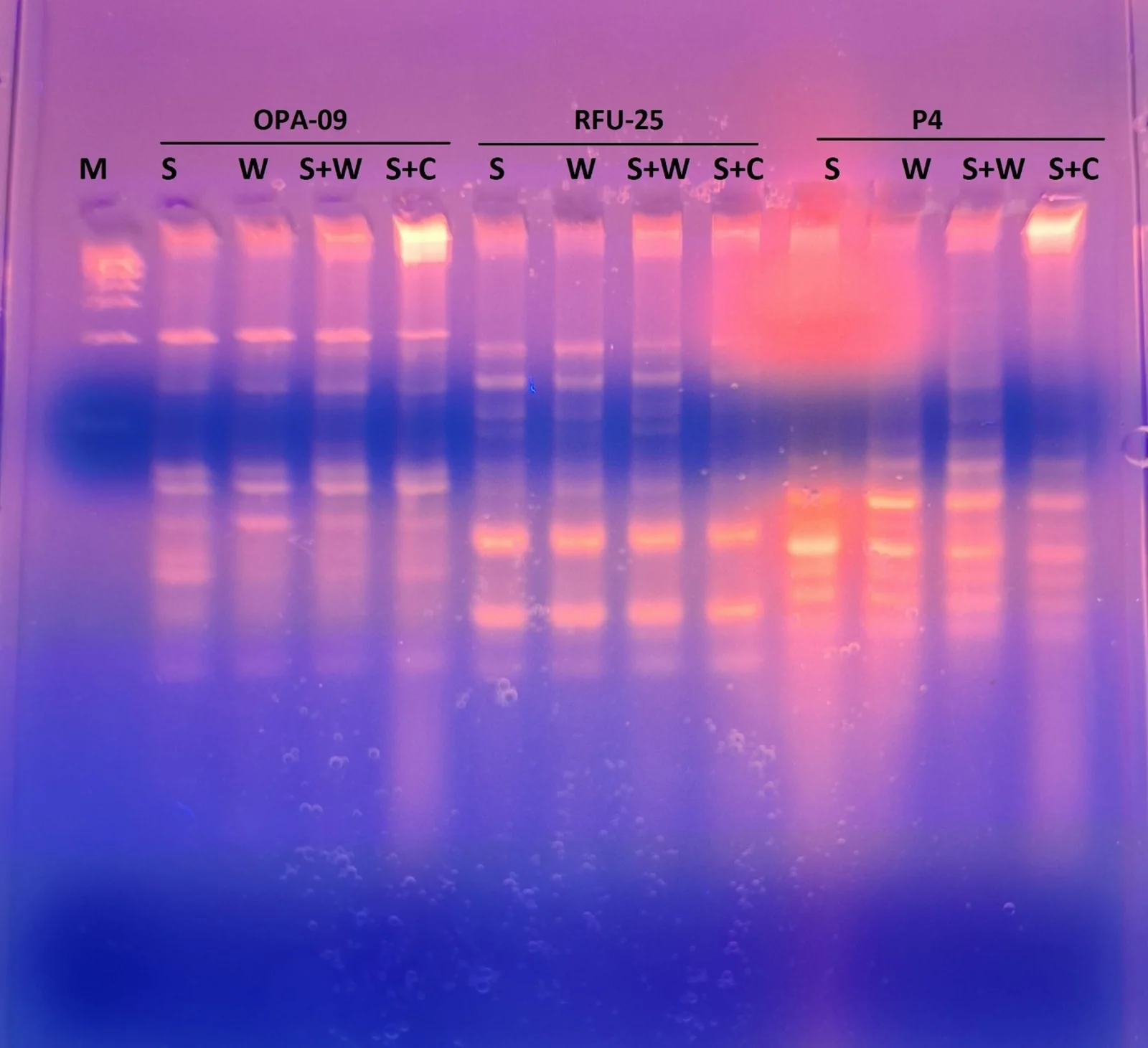
Gel profile of RAPD-PCR for barley species cultivated in different soils with 3 decamers primers. (M: 100 bp DNA ladder, S: clay soil, W: sawdust soil, S + W: clay combined with sawdust, S + C: clay combined with nanocellulose).

The RAPD-PCR gel profile (Table 3; Fig. 8) exhibits diverse banding patterns among several soil treatments, characterized by differences in band intensity, presence or absence, and fragment sizes. The clay combined with nanocellulose (S + C) and sawdust (W) exhibits distinct polymorphic bands in contrast to pure clay (S), indicating that soil additions may provoke genetic or epigenetic alterations in barley. The emergence of new bands in S + C may signify alterations in stress-responsive genes induced by nanocellulose, whereas the lack of certain bands in W could suggest sawdust-mediated repression of specific genomic areas. The 100 bp DNA ladder (M) verifies correct fragment sizing; nonetheless, faint or smeared bands in certain lanes (e.g., S + W) may indicate PCR artefacts or partial DNA degradation. The total polymorphism percentage from RAPD-PCR was 22.5%.

**Fig. 9:**
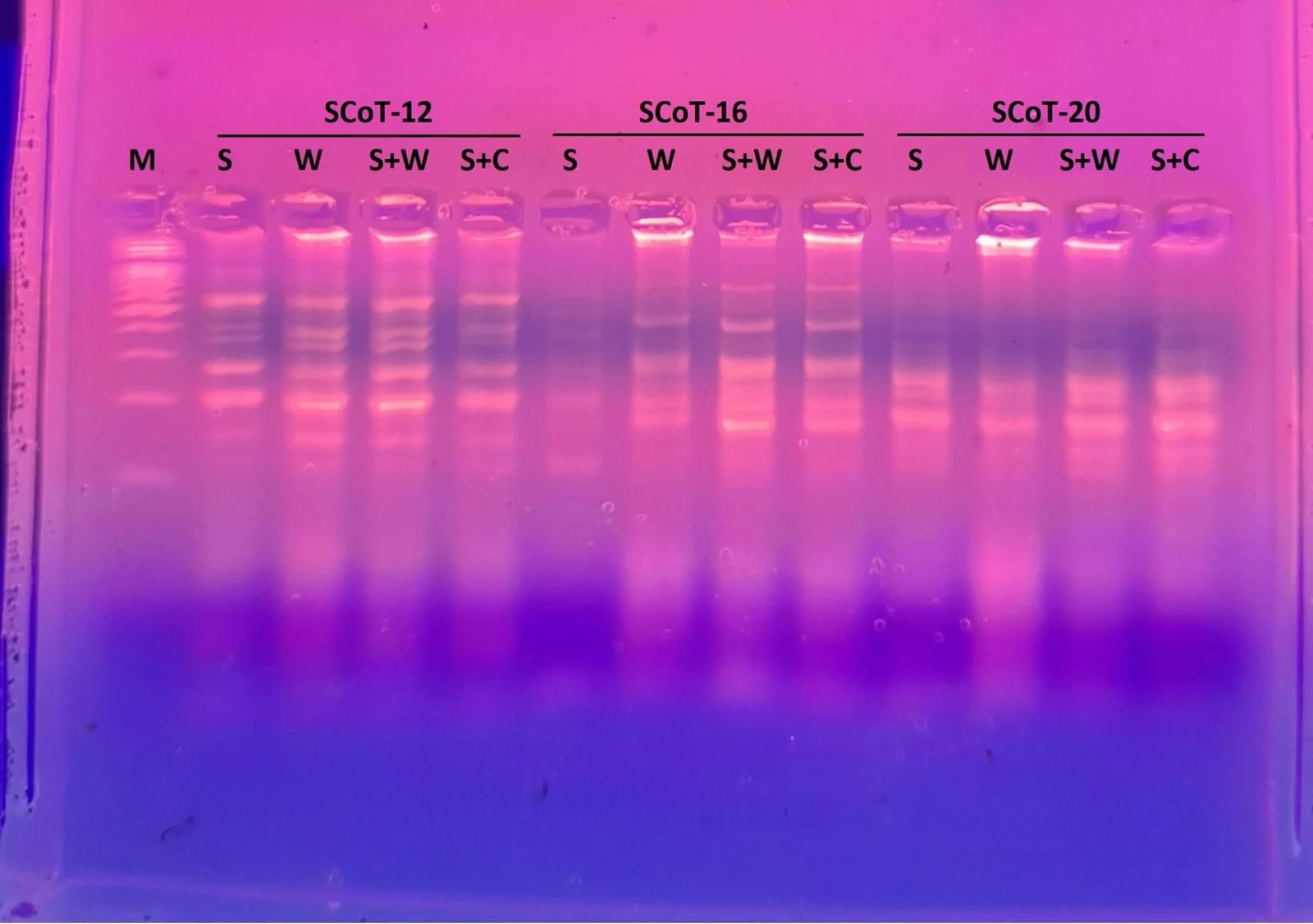
Gel profile of SCoT-PCR for barley species cultivated in different soils with 3 SCoT primers. (M: 100 bp DNA ladder, S: clay soil, W: sawdust soil, S + W: clay combined with sawdust, S + C: clay combined with nanocellulose).

The SCoT-PCR gel (Table 4; Fig. 9) shows bands that are consistent but not as variable, which is in line with SCoT’s lower polymorphism rate of 13.7%. SCOT-20 makes bands that are more variable (20% polymorphism), which could mean that it targets functional genes that are changed by soil treatments. The clay + sawdust (S + W) mix has banding patterns that are in between the two extremes. This could mean that both substrates influence each other. The small number of polymorphic bands across primers, on the other hand, shows that SCoT’s gene-targeted method might miss neutral changes that RAPD picks up. Both gels show how the molecular profile of barley changes depending on the type of soil it grows in. Nanocellulose and sawdust have the most effects. The total polymorphism percentage from SCoT-PCR was 13.7%.

The RAPD and SCoT results are now interpreted as indicating molecular polymorphisms or changes in DNA banding patterns associated with different soil amendment treatments.

**Table 3:** Gel banding pattern of RAPD-PCR for barley species cultivated in different soils with 3 decamers primers.

No.	Primer name	Primer sequence	GC%	Tm	Total bands	Total polymorphic bands	Polymorphism %
1	OPA-09	5’- GGGTAACGCC − 3’	70	37.4	10	2	20
2	Rfu-25	5’- CCGGCTGGAA − 3’	70	39.8	10	0	0
3	P4	5′- GAGCGCCTTG − 3’	70	38.8	12	3	25
Total					32	5	22.5

**Table 4:** Gel banding pattern of SCoT-PCR for barley species cultivated in different soils with 3 primers.

No.	Primer name	Primer sequence	GC%	Tm	Total bands	Total polymorphic bands	Polymorphism %
1	SCoT-12	5’- ACGACATGGCGACCAACG − 3’	61	58.4	9	1	11.11
2	SCoT-16	5’- ACCATGGCTACCACCGAC − 3’	61	57.3	10	1	10
3	SCoT-20	5′- ACCATGGCTACCACCGCG − 3’	66	60.8	10	2	20
Total					29	4	13.7

### Bioremediation application

**Fig. 10:**
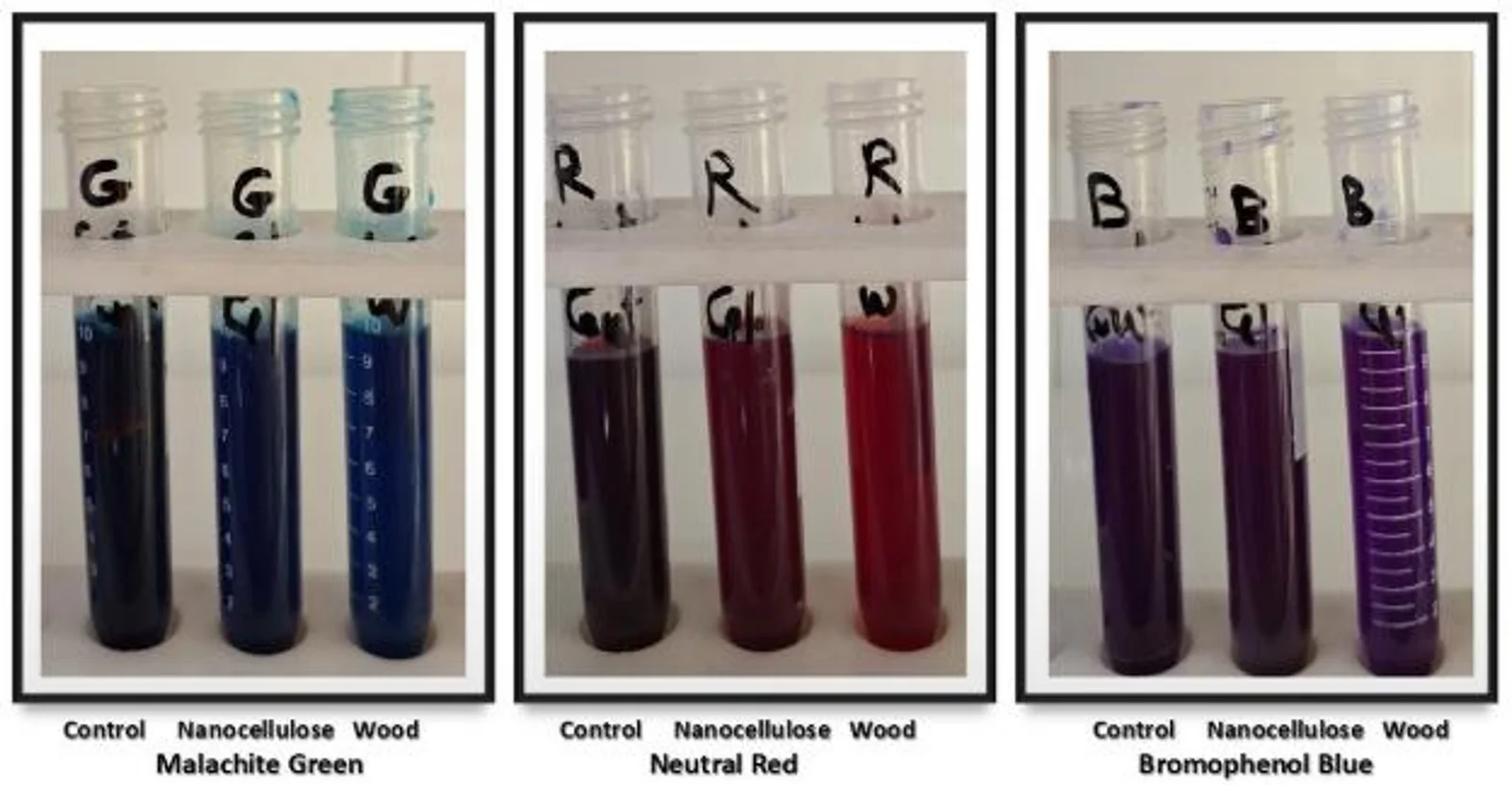
Decolorization of synthetic colors utilizing nanocellulose and wood sawdust as adsorbents.

**Fig. 11:**
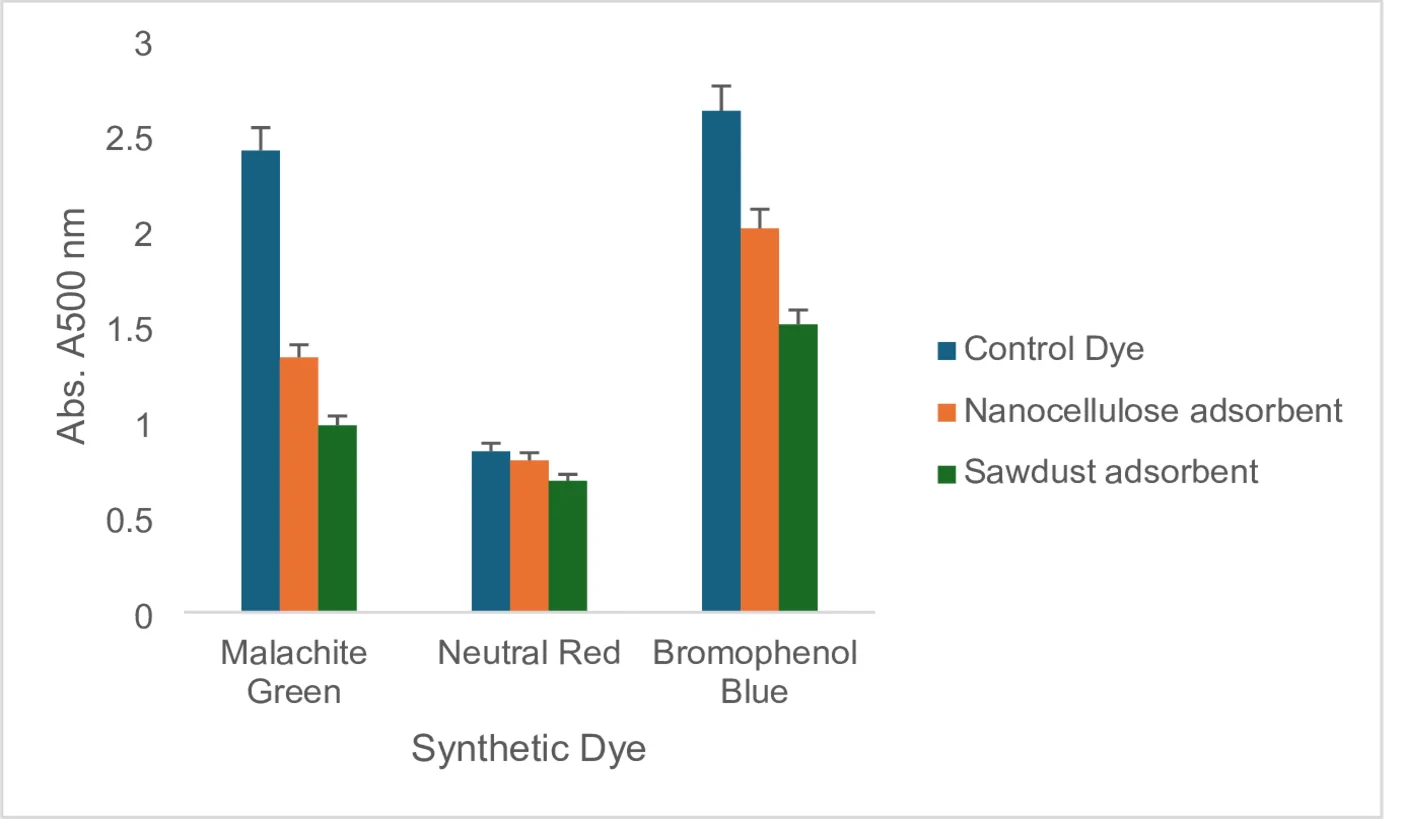
Absorbance of synthetic dyes adsorbent on surface of nanocellulose and sawdust.

Figure 10 and its graphical representation in Fig. 11, illustrated a comparative visual evaluation of dye decolorization efficacy utilizing nanocellulose, wood sawdust, and control (absence of adsorbent) for three synthetic dyes: Malachite Green, Neutral Red, and Bromophenol Blue. In all three sets, the control tubes exhibited the darkest coloration, signifying no removal occurred. Malachite Green exhibited significant adsorption by both nanocellulose and wood, with the wood treatment marginally surpassing nanocellulose in pigment reduction, as indicated by the visibly lighter solution. In the Neutral Red group, nanocellulose demonstrated superior decolorization compared to wood, evidenced by the pronounced fading of the dye, indicating effective interaction between cellulose functional groups and cationic dye molecules. Bromophenol Blue exhibited moderate impacts from both nanocellulose and wood, although neither resulted in significant color loss, indicating a restricted affinity or sluggish adsorption kinetics for this dye. The findings demonstrate that nanocellulose and wood-derived materials possess significant bioremediation potential, especially for cationic dyes, attributable to their elevated surface area, porosity, and functional groups such as hydroxyl and carboxyl, which enhance dye adsorption.

### Wound healing medical application

**Fig. 12:**
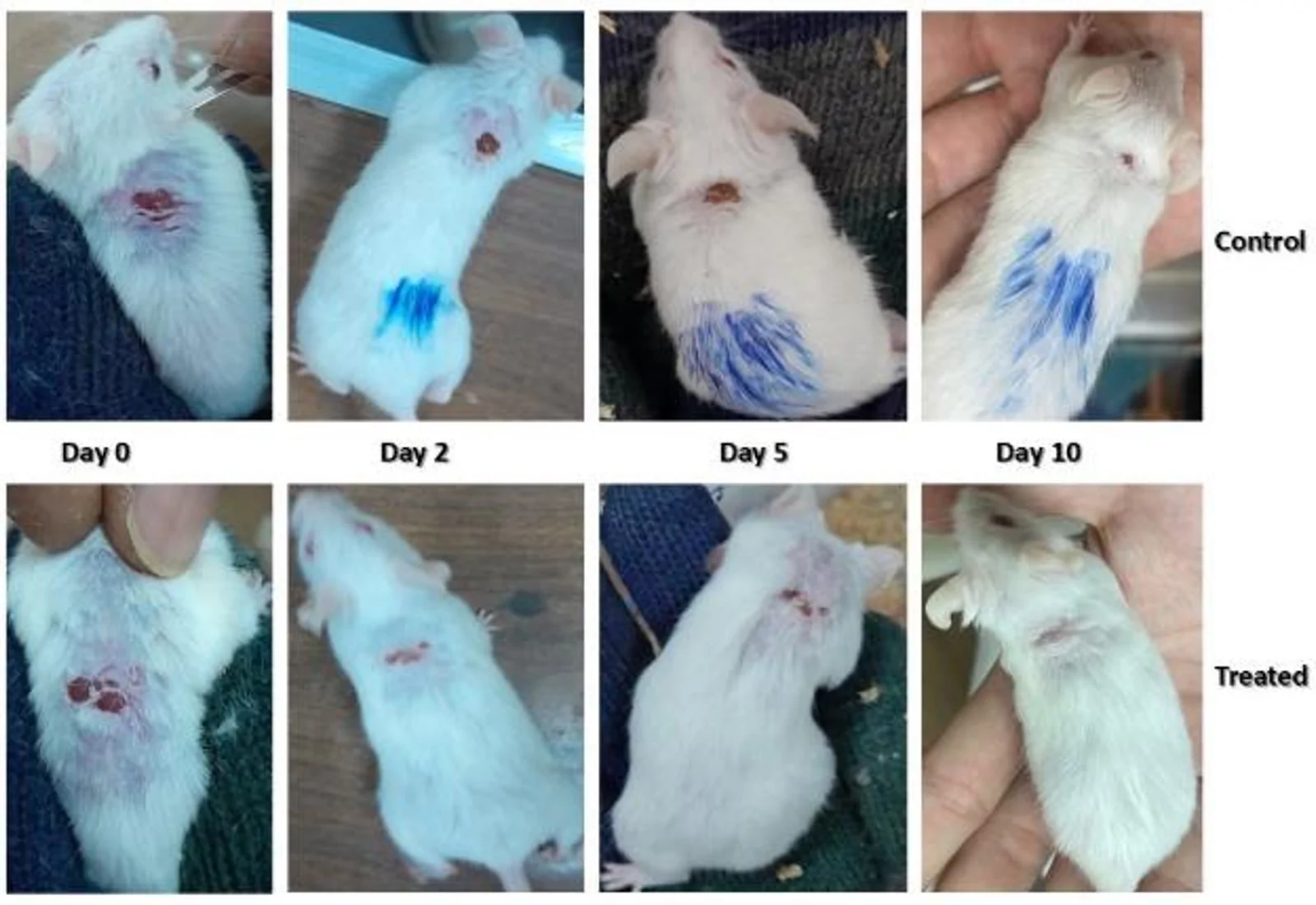
Wound healing progression in mice over 10 days with and without nanocellulose treatment.

Figure 12 showed how the wounds healed over 10 days in mice. The bottom row shows a group that was treated with nanocellulose, whereas the top row shows a control group. From Day 0, both groups have wounds that are around the same severity, which shows that the injuries were the same from the start. By Day 2, the control group has some scabs, but the treated group already shows signs that the inflammation is going down and the wound margin is getting smaller. The control wound is still clearly darker and more inflammatory on Day 5, but the treated wound is much smaller, less red, and has evident epithelial regeneration. The nanocellulose-treated wound is almost closed by Day 10, with hair coverage restored and only a little scar. In comparison, the control group still has a prominent scab and incomplete closure. These findings imply that nanocellulose speeds up the healing of wounds. This is probably because it is biocompatible, holds moisture well, and may help cells grow and move. The fact that the treated group’s wounds were getting better shows that nanocellulose could be a good natural biomaterial for skin regeneration.

**Fig. 13:**
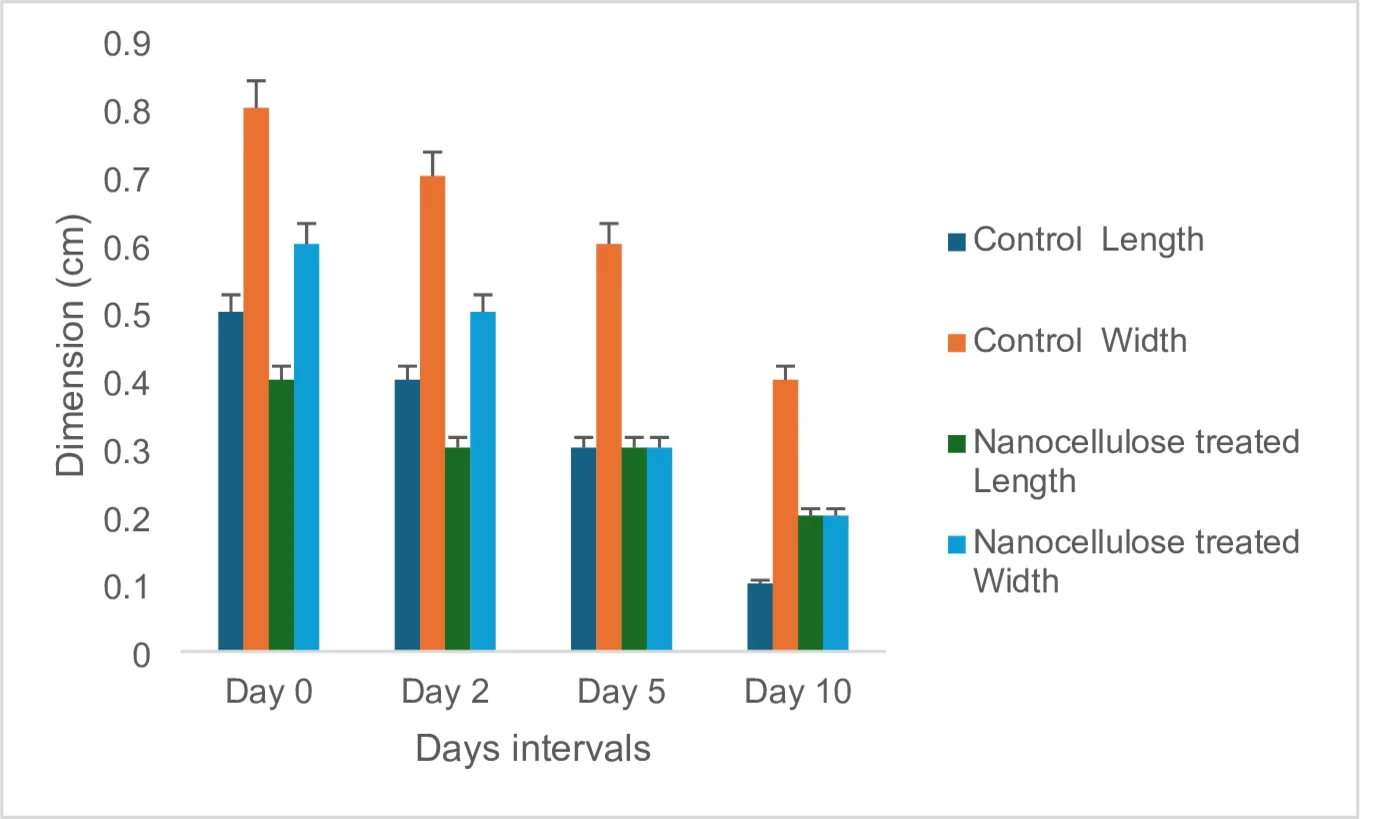
Diameter measurements of wound healing progression in mice over 10 days with and without nanocellulose treatment.

Figure 13 showed the graphical representation of comparison of how wounds in mice healed over 10 days, with and without nanocellulose therapy, based on measures of length and width. On Day 0, the wounds in both groups were about the same size, however the treated group’s wounds were a little smaller at first (0.4 × 0.6 cm) than the control group’s (0.5 × 0.8 cm). By Day 2, the wounds in both groups had shrunk, but the treated group (0.3 × 0.5 cm) had shrunk more than the control group (0.4 × 0.7 cm), showing that they were healing faster in the start. The nanocellulose-treated wounds were 0.3 × 0.3 cm on Day 5, which was more symmetrical and faster than the control wounds, which were still 0.3 × 0.6 cm. The treated group showed almost complete healing by Day 10 (0.2 × 0.2 cm), while the control wounds were still 0.1 × 0.4 cm and were closing more slowly. These data show that nanocellulose speeds up wound healing a lot, perhaps by making the surface moist and bioactive, which helps tissue regrow and lowers inflammation.

## Discussion

The results clearly show that adding sawdust and nanocellulose to the soil has a big effect on how barley grows. The clay + cellulose (S + C) treatment always produced the longest roots, leaves, and shoots, beating both the sawdust-only and control (clay-only) treatments. This is in line with research that shows that nanocellulose or nanofiber-based hydrogel composites improve soil structure, water retention, and nutrient availability, all of which help roots grow and shoots develop^24^. Nanocellulose’s high surface area and hydrogel-like behavior helps keep moisture around the root zone, which makes it easier for plants to take in water and move nutrients, as seen in studies of peas and other crops.

The clay + sawdust (S + W) amendment also increased the number of roots and shoots compared to the control with only clay. This is probably because organic matter helps the soil more porous and lets air in. But the effect wasn’t as strong as it was with cellulose. There is evidence that adding sawdust to some crops (such maize and wheat) can help them hold more water and grow more biomass. However, too much sawdust can temporarily stop nitrogen from moving. This is the same here, where S + W increased morphological features but wasn’t as effective as S + C. This is probably because nanocellulose provides similar structural benefits without taking away nitrogen. Barley cultivated in a medium made exclusively of sawdust showed slower growth in most areas, especially in the length of the leaves and shoots. This is probably because an organic-only substrate doesn’t have enough nutrients and doesn’t get enough air. Sawdust alone doesn’t have the minerals it needs, therefore it may not be able to get nutrients until it breaks down^25^.

The physiological findings demonstrate that the clay and sawdust treatment markedly improve many biochemical features in barley, indicating a synergistic advantage from the amalgamation of organic matter with mineral-rich soil. The chlorophyll index (1.5) went down in sawdust-only soil, but it still had a lot of protein and flavonoids, probably as a defense mechanism against stress caused by nutrient imbalance or nitrogen immobilization, which is common in woody organic substrates^26^. Clay + nanocellulose treatment had modest levels of physiological parameters, which supports its role in enhancing water retention and keeping metabolic functions going, but it didn’t reach the same levels as organic additions.

The molecular investigation of barley grown in several soil types: clay, sawdust, nanocellulose, and their combinations—utilizing RAPD-PCR and SCoT-PCR demonstrated unique patterns of genetic diversity affected by soil composition. These results correspond with earlier research indicating that soil amendments might elicit genetic and epigenetic modifications in plants, influencing stress responses and growth characteristics^27,28^. The observed RAPD and SCoT polymorphic banding patterns suggest molecular variation associated with different soil amendment treatments. However, these markers do not distinguish between genetic mutations, epigenetic modifications, or PCR-related polymorphisms. Therefore, the observed differences should be interpreted as molecular fingerprints reflecting treatment-associated genomic variability rather than direct evidence of heritable genetic or epigenetic changes.

The 22.5% polymorphism detected across RAPD primers indicates moderate genetic diversity across barley samples. The lack of polymorphism with Rfu-25 may suggest primer-specific constraints, as RAPD markers exhibit great sensitivity to PCR conditions^29^. The 20–25% polymorphism observed with OPA-09 and P4 indicates that specific soil treatments, such as nanocellulose or sawdust, may provoke observable variations in DNA sequences or methylation alterations with these primers.

Previous research indicates that nanocellulose can modify root shape and nutrient absorption^30^, potentially elucidating the distinctive banding patterns observed in S + C (clay + nanocellulose) samples. Likewise, sawdust (W) may instigate microbial interactions that affect barley’s genetic expression^31^, perhaps resulting in polymorphic bands. Nonetheless, the limited reproducibility of RAPD necessitates the use of confirmatory approaches such as SCoT or SSR markers^32^.

SCoT markers, which focus on gene-coding areas, exhibited reduced polymorphism (13.7%) however shown greater biological significance in comparison to RAPD. The 20% polymorphism seen with SCoT-20 indicates that specific soil treatments, such as nanocellulose, can affect stress-related genes, given that SCoT primers often amplify regions associated with abiotic stress responses^33^.

The treatment with clay and nanocellulose (S + C) may have altered the expression of genes involved in cell wall production or osmotic control, considering the established impact of nanocellulose on plant cell architecture. Simultaneously, sawdust (W) may have activated lignin-modifying enzymes owing to its elevated organic content, elucidating the polymorphic bands observed in SCoT profiles^34^.

Our results show that both wood sawdust and nanocellulose can lower the amount of synthetic dyes, but they work better on some types of colors than others. Both materials showed visible decolorization for Malachite Green, a cationic triphenylmethane dye. Sawdust worked a little better than the other material. This is in line with earlier research that found high removal rates utilizing untreated wood waste. For example, neem sawdust adsorbed malachite green through favorable Langmuir-type interactions with maximal capacities of about 4–83 mg/g. This shows that it is a good low-cost biosorbent. The dye’s cationic character probably makes it more likely to stick to the negatively charged cellulose surfaces and lignocellulosic matrix^35^.

Nanocellulose worked better than sawdust for Neutral Red (which is also cationic). This is probably because it has a larger surface area, a nanofibrillar structure, and a lot of functional groups (like hydroxyl and carboxyl) that help it interact with dye molecules. Batch testing has revealed that nanocellulose made from agricultural waste like sugarcane bagasse can get rid of up to 98% of malachite green. This suggests that neutral red could work just as well^36^.

On the other hand, both examples had only a small amount of Bromophenol Blue removed. This could be because its anionic structure doesn’t work as well with the adsorbents utilized. This shows how important charge properties are in dye-adsorbent systems^37^.

The current study clearly shows that putting nanocellulose on wounds directly speeds up the healing process in mice. Wounds that were treated with nanocellulose got smaller over the course of ten days, going from 0.4 × 0.6 cm to 0.2 × 0.2 cm. In contrast, the control group that didn’t get any treatment only got smaller by 0.1 × 0.4 cm. This faster contraction suggests better re-epithelialization and dermal remodeling, perhaps because the material holds onto water well, is biocompatible, and has a hydrogel structure that keeps moisture in, which creates the best conditions for healing.

These results are in line with earlier research that showed that nanocellulose hydrogels speed up wound healing by keeping the wound beds moist, promoting the growth of fibroblasts, and lowering inflammation. For example, bacterial nanocellulose coverings helped full-thickness wounds heal faster in living organisms and helped new blood vessels and tissue grow. Cellulose nanofibril-based hydrogels have also shown great antibacterial activity and wound closure rates, which shows that they can operate as both moisture-retaining scaffolds and barriers to microbes^38,39^.

When plant cellulose is present in the form of a hydrogel with nanofibrils, it can promote wound healing on its own, without the need for additional ingredients. Because of its great capacity to absorb liquids and create translucent films, wood-derived nanofibrillar cellulose (NFC) has been explored for use in wound dressing applications^39^.

Wood-derived cellulose crosslinked with calcium ions displayed haemostatic properties, particularly when supplemented with kaolin or collagen. Furthermore, inflammatory response experiments with blood-derived mononuclear cells demonstrated the inert nature of nanocellulose hydrogels in terms of cytokine secretion and reactive oxygen species generation. Water retention studies demonstrated the ability of nanocellulose hydrogels to maintain a suitable moist environment for various types of wounds^9^.

The mode of action of the adsorption process between nanocellulose and the contaminant is mostly determined by the type of surface modification on nanocellulose. If the pollutant is positively charged, such as heavy metals or colours, nanocellulose with a negative charge aids its adsorption and removal. If the contaminant is oil with hydrophobic and oleophilic properties, the nanocellulose is changed by introducing hydrophobic groups with lower surface energy, which aids in adsorption to the nanocellulose^40^.

Nanocellulose is thought to be one of the best materials for wound dressing. This is due to its favorable physical, chemical, and biological qualities, which include chemical purity, good mechanical properties, and water-absorbing ability^41^. Mohamad^42^, proved with in vivo experiments in mice revealed that BC/AA hydrogels loaded with cells had the greatest acceleration on burn wound healing, followed by hydrogel alone and the untreated group. On day 13 after wound coverage, the wound reduction percentages for the hydrogel loaded with cells, the pure hydrogel, and the control untreated group of animals were approximately 77%, 72%, and 65%, respectively. The transferred cells are believed to aid in commencing the wound healing process, where fibroblasts play a part in building the granulation tissue and keratinocytes help in re-epithelialization.

## Conclusion

The current work introduced a sustainable solution for wood waste (sawdust) to be turned into ecofriendly products. The study manipulated four applications for sawdust and nanocellulose in four fields: water conservation, crop production, bioremediation and medical applications (it shows how useful it could be in a clinical setting).

In conclusion, adding sawdust to clay soil makes a better soil conditioner that holds more water, encourages roots to grow, and makes shoots grow faster than organic-only substrates. This is based on biomaterials that can help crops grow better, especially when there isn’t much water. These results encourage the use of sawdust and nanocellulose-amended soils in sustainable farming encourage the use of sawdust and nanocellulose-amended soils in sustainable farming on a larger scale, especially to improve how efficiently cereals like barley utilize water and how they grow.

In short, both wood waste and nanocellulose show a lot of potential for cleaning up dyes. Wood sawdust is a cheap and easy way to get cationic dyes like malachite green, but nanocellulose is more efficient and flexible, especially for neutral dyes like neutral red. To get the best results, future studies should investigate surface functionalization (such carboxylation and amination), kinetic and isothermal modelling, and the possibility for regeneration. The results clarified that the dye-removal are proof-of-concept under controlled laboratory conditions and that real-world wastewater complexity (competing ions, organic matter, variable pH) has not been tested.

Overall, our findings support the idea that wood-derived nanocellulose is a powerful biopolymer for healing wounds. The fact that the material reduced the size of the wound by more than 75% compared to the control group shows how useful it could be in a clinical setting. Future research should look into nanoscale tissue integration, inflammatory biomarkers, and long-term remodeling to get a better understanding of how nanocellulose-based dressings work.

## Data Availability

The datasets used and/or analyzed during the current study available from the corresponding author on reasonable request.
